# Osteocalcin: A Multifaceted Bone-Derived Hormone

**DOI:** 10.1146/annurev-nutr-061121-091348

**Published:** 2023-08-21

**Authors:** Gerard Karsenty

**Affiliations:** Departments of Genetics and Development, Vagelos College of Physicians & Surgeons, Columbia University, New York, New York, USA

**Keywords:** bone, osteocalcin, undercarboxylation, energy metabolism, danger repellent

## Abstract

Together, loss- and gain-of-function experiments have identified the bone-derived secreted molecule osteocalcin as a hormone with a broad reach in rodents and primates. Following its binding to one of three receptors, osteocalcin exerts a profound influence on various aspects of energy metabolism as well as steroidogenesis, neurotransmitter biosynthesis and thereby male fertility, electrolyte homeostasis, cognition, the acute stress response, and exercise capacity. Although this review focuses mostly on the regulation of energy metabolism by osteocalcin, it also touches on its other functions. Lastly, it proposes what could be a common theme between the functions of osteocalcin and between these functions and the structural functions of bone.

## PREAMBLE

There is a stark contrast between the general perception, among laypersons and scientists alike, of what bone is or does and what it is actually needed for. If anything, this contrast has become even more pronounced as we have progressed in our understanding of what the biology of bone encompasses. It is not an exaggeration to write that for anybody who is not studying them, bones are merely an assembly of calcified tubes, an inert structure that is lifeless or almost dead. It may, therefore, not be too surprising that—further extending and strengthening this negative perception of bone—in any culture in the world, the symbol of death or of the danger of death is bone. And yet, by all accounts, the physiology of bone is defined exactly as the opposite: It is the physiology of organs that is essential to protect from death.

Let us take the more widely accepted image of bone, the one of an assembly of calcified tubes, an image that is not totally inaccurate. As a calcified scaffold, bone protects internal organs from the danger of damage followed by death in the case of trauma. As a calcified, rigid structure, bone also allows for walking and running, the latter being another way to escape mortal danger. One more vivid example of this functional identity of bone as a danger repellent is provided by the presence of three small bones in the middle ear. These bones are necessary for hearing. In the wild, deafness has always, and continues to, put animals at great risk of death. Hence, under this light, even the most traditional functions of bone contradict its perception as an inert scaffold that may become medically interesting only after menopause, when osteoporosis develops. Instead, it seems to be, because of its structural functions, an organ needed to escape lethal dangers. Even if this is only one facet of the biology of bone, it is an interesting one to consider because it begs the following question: If one hypothesizes that bone may have functions other than its structural ones, could these putative novel functions also be implicated in the role of bone as a danger repellent? This is the overarching question that looms behind the entire work presented in this review, a question we come back to in the concluding remarks of this article.

## INTRODUCTION

Among all tissues of the body, bone distinguishes itself by the unique coexistence of two features. It is a mineralized tissue, a feature it shares with the tooth (2, 16, 55). But perhaps more relevant to this review article is its other specific feature: Bone is the only tissue in the body that contains a cell type, the osteoclast, whose sole function is to actively destroy or resorb the host tissue, that is, the mineralized bone, and it does this throughout life (52). Of course, many tissues, not only bone, regenerate during life, but only bone goes through a cell-mediated process of active self-destruction. Furthermore, constantly following this phase of self-destruction that occurs daily in hundreds of locations, there is de novo bone formation by another bone-specific cell type, the osteoblast (14). This perpetual alternation of destruction and formation that occurs in all bones from birth to death is called bone modeling during childhood and bone remodeling once longitudinal growth has stopped (24).

Bone modeling is necessary for the longitudinal growth of the skeleton; as for bone remodeling, it was originally meant to repair micro- and macrodamages, in other words, fractures. It is only during modern times that it has become the physiological process affected by osteoporosis.

This alternation from the cradle to the grave of self-destruction followed by de novo formation is unique to bone and explains why, since its origin, bone biology has been preoccupied with one fundamental question: What regulates bone resorption and/or bone formation? In other words, what are the molecules, hormones, or neurotransmitters that signal into osteoclasts and osteoblasts? To put it in another way: Of which and how many signals is bone the target? While this biological paradigm has been fertile beyond expectation both in biological and therapeutic terms, it has de facto led to the assumption that bone is only a recipient of molecular influences and does not itself dispense any influence on the rest of the body. A broader view of bone biology suggests that this conception of bone biology as a one-way street is incomplete and, in a way, deprives bone of a wealth of interesting aspects.

The active destruction of the mineralized bone is a costly process in energetic terms. Likewise, the synthesis, secretion, and mineralization of the fibrils of collagen that are the main constituents of the bone extracellular matrix (ECM) are, because of the size of these fibrils and the large quantity of them that is produced, an energetically expensive process. As it is the case for the energetic cost of any other physiological processes, the energetic cost of bone modeling and remodeling is proportional to the area covered by the organ in which these processes occur. There are more than 200 bones in the human body, and together they cover a large surface. Hence, bone modeling and remodeling likely must be energetically costly processes. If one assumes that they are, then it is logical to consider that there should be a relationship between bone modeling/remodeling and energy metabolism to avoid too much or too little energy being allocated to bone modeling and remodeling.

The very nature of bone as a mineralized tissue makes it difficult to rigorously assess the energetic cost of bone modeling and remodeling in vivo in any model organism. However, answers to this question are apparent from clinical medicine. Indeed, clinical observations provide direct support to the notion that energy metabolism and bone (re)modeling are functionally linked. For instance, regardless of its cause, the lack of ingestion of food, that is, the lack of energy intake, results in an arrest of longitudinal growth in children (35, 54). In adults, starvation, regardless of its cause, also results in loss of bone mass (1, 49). In other words, energy uptake impacts bone biology. Another clinical observation, albeit totally unrelated to energy metabolism but of great importance if one tries to look at bone biology through a different prism, is that the arrest of gonadal functions in both sexes results in rapid and often severe bone loss. One way to combine these apparently distinct clinical observations into a single biological hypothesis that would be experimentally testable in vivo is to propose that there may be a coordinated regulation that is endocrine in nature (since the organs involved are not close to each other) of energy metabolism, bone mass, and reproduction (48). This represents the first conceptual framework that was proposed to test why and how bone could influence some cardinal organismal physiological functions that all take place outside the bone. Over time, this conceptual framework has proven to be not only extremely fertile in broadening the definition of bone physiology but also, as we present, too restrictive.

The tripartite hypothesis mentioned above comes with some limitations that should be viewed as guiding tools. The first one is that the very existence of bone justifies the existence of this hypothesis. Hence, if this hypothesis is correct, the hormones responsible for this coordinated regulation should appear during evolution with bone, not before. If we look at the actual functions of molecules, this is undoubtedly the case for leptin, which was the first hormone link identified between energy metabolism (appetite and energy expenditure), bone modeling and remodeling, and reproduction. A second guiding constraint or implication of this hypothesis is that, to comply with a fundamental principle of endocrinology, if it is true that bone is a recipient of endocrine inputs, then bone should also be an endocrine organ regulating at least aspects of energy metabolism and reproduction. In other words, what this hypothesis proposed to do was to reverse the paradigm on which the entire field of bone physiology was originally built. Last but not least, since it has to do with the biology of bony vertebrates, this hypothesis should be testable in the mouse, as the molecules involved can only be present in bony vertebrates. It is precisely the testing of this hypothesis that, after a long detour through fat and the brain, led to the discovery of osteocalcin as a bone-derived hormone.

## OSTEOCALCIN IS A MOLECULE IN SEARCH OF AN IDENTITY

Osteocalcin is, in more ways than one, a veteran of bone biology (12). It is an abundant protein of the bone ECM that was purified in the late 1970s at the height of the protein chemistry era of bone biology (53). It has remained since then an enigma of bone biology for several reasons, not the least of them being that for a long time, this protein that is so abundant [it is the 10^th^ most abundant protein in the body (20)] had no demonstrable function in vivo. Osteocalcin is a small protein (49 amino acids long in humans and 46 amino acids long in rodents) with a molecular weight of 5 kDa that is synthesized by osteoblasts. Osteocalcin is also a secreted protein that is carboxylated on three glutamate residues. Carboxylation is a posttranslational modification operated by the enzyme gamma carboxylase in the presence of vitamin K as a cofactor (17). This posttranslational modification confers to proteins a high affinity for calcium and phosphate ions and mineral structures in general (17). This modification is the one that is seen in some coagulation factors and in matrix Gla protein, a protein distantly related to osteocalcin that is an inhibitor of ECM mineralization in cartilage and arteries (20). Given this feature, the other name of osteocalcin is bone Gla protein (BGP). In the premouse genetic era, because of its abundance in the bone ECM and its ability to bind minerals through its GLA residues, osteocalcin was generally suspected to be a regulator of ECM mineralization. This contention, however, could never be verified beyond microscopic infraclinical features in vitro, let alone in the mouse. Moreover, what was immediately surprising about osteocalcin as a protein was that it seemed to have two forms. One is the carboxylated protein, which is the most abundant noncollagenous protein of the bone ECM. In fact, at mole per mole, there is as much osteocalcin, fully carboxylated, in the bone ECM as there are collagen molecules (20). A second form is an undercarboxylated osteocalcin that is present in the general circulation in the nanograms per milliliter range, that is, the range seen for many hormones (20).

Other molecular aspects of osteocalcin that were initially overlooked suggested that this molecule could be something other than a structural component of the bone matrix and possibly a hormone. The first one is that the genes encoding osteocalcin are expressed only in osteoblasts (18, 20). As a matter of fact, until now, *Osteocalcin* remains one of the very few genes that are osteoblast specific in their expression. This could, of course, be the case for a molecule regulating bone ECM mineralization, but it could also be the case for a peptide hormone since most of them are encoded by cell-specific genes. Consistent with the latter hypothesis, the osteocalcin genes, one in humans, two in mice, encode for a long preproprotein that is subsequently cleaved twice to give rise to mature osteocalcin that is secreted outside the cell (20). A cell-specific gene encoding a prepropeptide that is subsequently cleaved and present in the general circulation in the milligram per milliliter range is not always, but most often, a gene encoding a peptide hormone (10, 29). This hypothetical endocrine identity of osteocalcin came to bear when a traditional bone-centric view of osteocalcin failed to uncover any of the tangible anticipated bone mineralization functions of osteocalcin in vivo (44). Indeed, despite the fact that osteocalcin is the most abundant noncollagenous protein of the bone ECM, mice lacking osteocalcin had an overall normal skeleton with only microabnormalities of bone mineralization that appeared late in their life (5, 13). The high bone mass they experience also appears relatively late in life and may, therefore, be a secondary event (13). This apparent negative result raised a more profound question: Why would evolution have invented a gene that is cell specific and encodes such an abundant protein for no functional purpose?

One way to answer this question, if no function for osteocalcin in the bone can be inferred from the analysis of *Osteocalcin*-deficient mice, may simply be to look at the other face of osteocalcin, that is, at the fact that it is a circulating molecule that looks very much like a hormone, and to ask, for the sake of leaving no stone unturned and of broadening bone biology, whether some or all functions of osteocalcin may take place outside the skeleton? This is where the biology of osteocalcin met, admittedly serendipitously, the hypothesis that bone mass, energy metabolism, and reproduction should be coordinately regulated by endocrine means. It did so because the only overt phenotype one could reliably observe in the *Osteocalcin*-deficient mice was that even though their body weight was normal, there was a marked increase in their visceral fat mass; furthermore, male *Osteocalcin*^−/−^ mice were relatively poor breeders. Both of these phenotypes appeared early in life, within a few weeks of birth, and certainly before the high-bone-mass phenotype in the *Osteocalcin*^−/−^ mice becomes detectable. This observation turned out to be the Ariadne’s thread that uncovered the broad biology of osteocalcin and as a result conferred to bone a richer identity—the identity of an organ, which is of both structural and endocrine nature. In so doing, bone fully assumed its identity as an evolutionary game changer when animals left the sea to live on land. It has collected in itself a series of functions, whether structural, mechanical, or endocrine, that are all needed to escape lethal dangers.

## A GROWING TOOL KIT TO STUDY OSTEOCALCIN

By definition, the in vivo study of the functions of any molecule is only as good as the quality and the number of tools available to study it. Fortunately, in the case of osteocalcin, these tools are numerous, growing in number, and, more importantly, from a biological point of view, unanimous in their description of the functions of this protein. This uniformity we allude to in this review article is part of another study currently being completed that is of fundamental importance for the purpose of this review.

As one would expect nowadays, most of these experimental tools are of genetic nature. Even though they are rightly so, as they are the cornerstone of modern endocrinology, genetic tools do not remove any value from the more classical pharmacological ones. The latter tools are important because pharmacological experiments can be conducted on animal species other than the mouse and more specifically in primates, broadening the significance of these findings.

Let us start with the genetic loss of functional tools. In addition to the original *Osteocalcin*-deficient mouse model we had generated (13), many more have since become publicly available. Indeed, three additional models of osteocalcin deficiency have recently been generated through a CRISPR-Cas9 approach; two of these models are available upon request, one of which was generated in the United States and the other in China (26, 46). These two publicly available models are the ones that were tested by us and that are mentioned in this review article. Importantly, these two models are maintained on distinct genetic backgrounds, mixed *C57/129Sv/eV* for the former and *C57/B6* for the latter, thus allowing one to ask how generalizable are the conclusions one draws from the study of various mouse strains of *Osteocalcin* inactivation. Moreover, and even though *Osteocalcin* is a cell-specific gene, a model of an osteoblast-specific deletion of *Osteocalcin* has also been generated and analyzed for at least one of the functions of this molecule (37).

The tool kit of loss-of-function models is broader than that. As the study of osteocalcin biology progressed, one, then two, then three osteocalcin receptors, all with distinct patterns of expression, were identified. This led to a remarkable increase in the number of loss-of-function mouse models available, since each of these three receptors, *Gprc6a, Gpr158,* and *Gpr37* has been deleted in the germ line and in a cell-specific manner in a wide variety of cell types (25, 32, 37, 41, 46, 58). These mouse models of cell-specific receptor deletion were used to demonstrate that these receptors were bona fide receptors for osteocalcin in three ways. The first one, of course, was to compare the phenotype of receptor-deficient mice with the phenotype of ligand-deficient mice. The second was to show that injection of osteocalcin in mice homozygous for the absence of a given receptor in a cell type of interest, whether it is myoblast or neuron, could not affect muscle or neuronal functions as it does in wild-type (WT) mice. The third way in which these receptor-deficient mouse models were used was by generating double-heterozygous mice lacking a single copy of *Osteocalcin* and a single copy of a given receptor in each cell type (25, 58). According to genetic epistasis, a pillar of Mendelian genetics, if osteocalcin is the bona fide ligand for a given receptor in a particular cell type, then compound heterozygous mice, lacking one allele of the ligand and one allele of the receptor specifically in this cell type, should have the same phenotype as mice lacking both copies of the ligand or of the receptor in this particular cell type (38). This closes the list of the mouse models of loss of osteocalcin signaling that are currently available. We should emphasize again that the field has been fortunate that, so far, the absence of osteocalcin results, as mentioned below, in the same phenotypic abnormalities in all *Osteocalcin^−/−^* mouse models testable, regardless of the mode of generation or the genetic background they are maintained on. In addition to these cell-specific loss-of-function mouse models, patients harboring a dominant-negative mutation in one of the osteocalcin receptors have been identified (36). Although this mutation is not cell specific, it is useful in so far as it extends some aspects of the biology of osteocalcin from mice to humans.

As mentioned above, the field also had access to the gain-of-function models without which the functional analysis of a hormone would be only halfway performed. Two of these models were of genetic nature; these are mice lacking the osteoblast and testis-specific protein tyrosine phosphatase (OST-PTP) in all cells or in osteoblasts only. OST-PTP is encoded by the gene *Esp* that is expressed in Sertoli cells of the testes and in osteoblasts only (9). In addition to these genetic models, classical pharmacological ones achieved by injecting exogenous osteocalcin have also been used because they provided a means to interrogate the biology of osteocalcin in nonhuman primates (7, 58). In this ongoing investigation of osteocalcin biology, loss- and gain-of-function models serve as an internal control of each other. What is important to emphasize in this review article is that it is the systematic and comparative use of loss- and gain-of-function models that have allowed the field as a whole to demonstrate that osteocalcin is necessary and sufficient to regulate the various physiological functions that are in its purview. We should add that the fact that exogenous osteocalcin is sufficient to improve the physiological functions it regulates is important for another reason that is of a more medical nature. Since circulating osteocalcin levels decrease steeply with aging, as do most physiological functions regulated by osteocalcin, the fact that exogenous osteocalcin could correct the deficit of some physiological functions that are stereotypical manifestations of aging in mammals suggests the possibility, which is currently being explored, that the biology of osteocalcin could be harnessed to fight some of the most frequent and crippling manifestations of aging.

## REGULATION OF ENERGY METABOLISM BY OSTEOCALCIN

### Energy Expenditure

**Figure 1:**
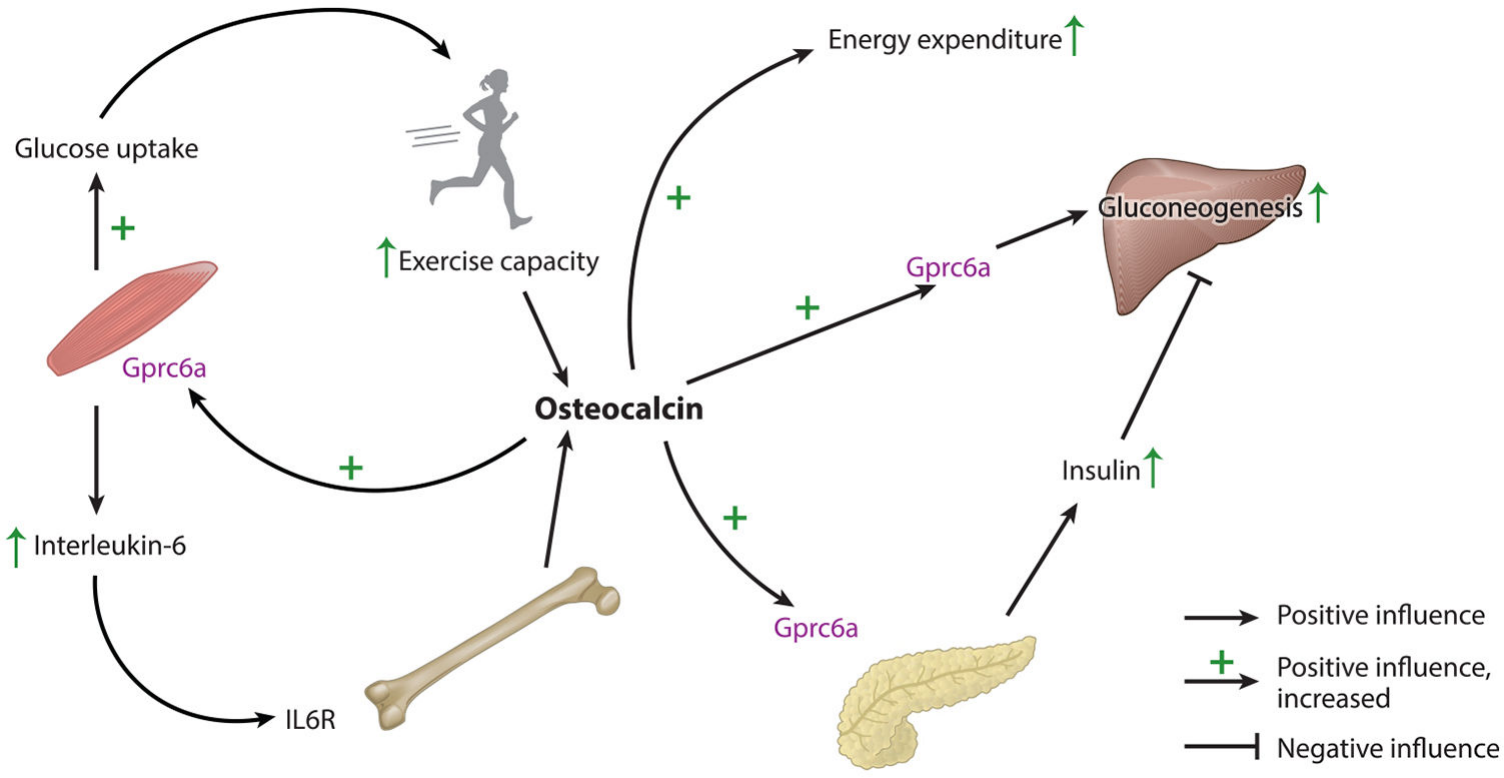
Regulation of energy metabolism by osteocalcin. Osteocalcin secreted from the bone regulates various aspects of energy metabolism. In the pancreas, it regulates insulin secretion and its action in different tissues. During exercise, osteocalcin is released from bone and increases glucose uptake in the muscle to increase exercise performance; muscle in turn secretes interleukin-6 in a positive feedback loop and increases secretion of osteocalcin from osteoblasts. Osteocalcin indirectly regulates adiposity by increasing energy expenditure.

As alluded to above, the first quantifiable phenotypic abnormality observed in *Osteocalcin*^*−/−*^ mice was an increase in inguinal fat mass. This was observed consistently in male and female mutant mice when they were sacrificed to reach their skeleton, regardless of the age at which the mice were sacrificed. This increase in visceral fat mass was not due to an increase in appetite, since appetite is normal in both *Osteocalcin*^*−/−*^ and *Esp*^*−/−*^ mice, the model of osteocalcin gain of function. Instead, this increase in visceral fat mass was caused by a decrease in energy expenditure in *Osteocalcin*-deficient mice (28). Surprisingly, since it was one of the first phenotypes identified in *Osteocalcin*^*−/−*^ mice, it is still in search of a molecular explanation, the reason for that being that none of the known receptors of osteocalcin are expressed in adipocytes. Nevertheless, this regulation of energy expenditure, which could be studied in three different mouse models of *Osteocalcin* inactivation and one genetic model of gain of function, was historically important because it was the first evidence that bone via osteocalcin was affecting organs other than the skeleton. This turned out to be the beginning of the demonstration that, in its endocrine capacity, bone, through various hormones, that is, osteocalcin, lipocalin-2, and possibly others, regulates energy metabolism (34) (Figure 1).

### Glucose Homeostasis at Rest and Running Exercise

This overt increase in fat mass observed in the *Osteocalcin*-null mice triggered a relatively simple metabolic analysis. The first abnormality that this metabolic study revealed was a marked increase in blood glucose levels, in all *Osteocalcin*^*−/−*^ mice analyzed and regardless of their sex and age, whether these levels were measured at random or after fasting. Conversely, and confirming that osteocalcin somehow regulates glucose homeostasis, *Esp*^*−/−*^ and *Esp*_*osb*_^*−/−*^ mice were hypoglycemic. This dysregulation of glucose homeostasis was due in part to a decrease in insulin secretion, since circulating insulin levels were lower in randomly fed *Osteocalcin*^−/−^ mice, regardless of their mode of generation, and higher in randomly fed *Esp*^*−/−*^ mice (28). As for a glucose-stimulated insulin secretion test, it showed the expected decrease in glucose-stimulated insulin secretion in *Osteocalcin*^*−/−*^ mice and an increase in *Esp*^*−/−*^ mice. Molecularly and histologically, there is a decrease in *Insulin* expression and insulin content in pancreatic β cells, a decrease in β-cell mass, and a decrease in the ability of β cells to proliferate, and, as a result, pancreatic islets are of smaller size in the absence of osteocalcin. In agreement with these observations, the absence of osteocalcin results in glucose intolerance as measured by a glucose tolerance test. It also induces a state of insulin resistance as measured by an insulin tolerance test. Importantly, and this is true for every osteocalcin target cell type so far, it is the uncarboxylated or undercarboxylated form of osteocalcin that is active in vitro in islets and β cells and that can enhance *Insulin* expression and secretion (28). Collectively, these data inferred that osteocalcin might directly or indirectly regulate the synthesis and release of insulin by the pancreatic β cells. In full agreement with this notion, one receptor of osteocalcin, *Gprc6a,* is expressed on pancreatic β cells, and its deletion specifically from these cells results in a decrease in β-cell mass and in *Insulin* secretion (42, 57). These observations were subsequently extended to humans. Indeed, in the most direct experiment, injection of exogenous osteocalcin in mice implanted with grafted human pancreatic islets increases human insulin production (50).

On the basis of these findings, the entire field, admittedly starting with us, began to view osteocalcin as an insulin secretagogue (59). While this view is correct, it is an incomplete one. Osteocalcin is more than that, since it also regulates glucose homeostasis in an insulin-independent manner. Pushing the paradox even further, in one organ, the liver, osteocalcin opposes the main function of insulin. While insulin opposes gluconeogenesis in the liver, osteocalcin favors this process by upregulating expression in the liver of the very same genes, *G6pa* and *Pepck*, that are downregulated by insulin signaling in hepatocytes (19). This progluconeogenic function of osteocalcin signaling in the liver also requires that osteocalcin signals through Gprc6a that is present in hepatocytes (43). This feature of osteocalcin, which was observed in all mouse models of *Osteocalcin* inactivation we could test, broadens substantially its biology and may explain why exogenous osteocalcin can never cause hypoglycemia even though it increases insulin secretion.

## REGULATION OF GLUCOSE METABOLISM BY OSTEOCALCIN DURING EXERCISE

In another organ muscle, and in another physiological setting, exercise, osteocalcin also acts independently and differently than insulin does. Revealing this feature of osteocalcin originated from the study of one hallmark of its biology. Circulating levels of osteocalcin abruptly rise in mice and in humans during endurance exercise, a physiological situation during which circulating insulin levels decrease. Exploring this feature of osteocalcin uncovered an endocrine regulation of endurance exercise and began to give a conceptual coherence to all known functions of osteocalcin (32).

Osteocalcin signaling in myofibers is needed for glucose uptake in myofibers during exercise in part because it favors, equally as well as the same molar amount of insulin would do, the translocation of the glucose transporter Glut4 to the cell membrane of myoblasts during an endurance exercise (21, 32). Unlike insulin, however, osteocalcin is catabolic in myofibers and promotes the breakdown of glucose into pyruvate and the utilization of pyruvate in the tricarboxylic acid cycle to produce ATP molecules that are needed for endurance exercise (32). This glucose uptake and breakdown function of osteocalcin in muscle during exercise is also mediated by Gprc6a, which is preferentially expressed in myofibers of oxidative muscle compared with those of glycolytic muscle. As expected, mice lacking Gprc6a in myofibers cannot uptake glucose during exercise and become hyperglycemic (32). Together, the functions of osteocalcin in the pancreas, liver, and muscle reveal a complex picture of the regulation of glucose homeostasis by this hormone. In one organ, the pancreas, osteocalcin is an insulin secretagogue; in another organ, the liver, osteocalcin has a function that opposes that of insulin; and in a third organ, the muscle, osteocalcin fulfills the glucose uptake function of insulin during exercise, that is, when circulating insulin levels drop. However, unlike insulin signaling, osteocalcin signaling in muscle is catabolic. In addition, and as one would expect from a hormone-promoting endurance exercise, osteocalcin signaling in myofibers through Gprc6a is needed, during exercise, for the uptake of fatty acids, their catabolism, and the transfer of acylcarnitine into the mitochondria to produce ATP molecules (32).

Besides these metabolic functions that increase exercise capacity, osteocalcin exerts another important function in myoblasts during exercise, a function that also contributes to increasing exercise capacity. Osteocalcin promotes the expression in these cells of interleukin-6 (*IL-6*) and the secretion in the general circulation of IL-6 (32). IL-6 is one of the first myokines ever described, and although it is known to increase muscle function during exercise, the mechanisms whereby it achieves this function in vivo were not fully understood (39, 40). As a matter of fact, the use of multiple models of ligand or receptor cell-specific gene deletion showed that when circulating osteocalcin levels rise during exercise, this increases the expression in and secretion by myoblasts of IL-6. IL-6 then signals in osteoblasts to increase osteoclast differentiation and thereby the release of bioactive osteocalcin. This signaling in osteoblasts is necessary for IL-6 to increase exercise capacity, as demonstrated by the observation that mice lacking the IL-6 receptor in osteoblasts experienced a profound decrease in their ability to perform endurance exercise (7). Remarkably, osteocalcin signaling in myofibers not only is necessary for muscle function during exercise and for exercise capacity but also is sufficient, in so far as a single injection of exogenous osteocalcin a few minutes before an endurance exercise starts to increase the ability of WT mice, but not of mice lacking *Gprc6a* in myofibers, to perform endurance exercise (32). This was seen in young mice but even more so in older mice that have low circulating osteocalcin levels (32). This work performed in genetically modified animals was extended in several studies to humans (30, 31).

## THE CROSS TALK BETWEEN OSTEOCALCIN AND INSULIN

Even if it is only one aspect whereby osteocalcin regulates glucose homeostasis, the role of osteocalcin as an insulin secretagogue raises an important novel question: Could it be that in a classical endocrine feedback loop, insulin regulates osteocalcin secretion? In other words, is bone an insulin target organ whose importance for the regulation of glucose homeostasis had not been previously appreciated?

The legitimacy of the latter question goes far beyond the biology of osteocalcin. Indeed, the inactivation of the insulin receptor in two of the most classical insulin target cell types, myoblasts and hepatocytes, does not result in the expected alterations of glucose homeostasis in animals fed normal chow (4, 6). Taken at face value, a logical and testable interpretation of these negative observations is that other organs may be insulin target ones and, as such, may contribute to the regulation of whole-body glucose homeostasis in animals fed a normal diet. As it could be legitimately hypothesized from regulation of insulin secretion by osteocalcin, in vivo, the osteoblast and the bone are such a cell type and organ, respectively.

Mice that lack the insulin receptor in osteoblasts develop hyperglycemia, glucose intolerance, and insulin resistance when fed a normal chow diet (15). A posteriori, this may not be too surprising, given the large surface covered by bones in the body and the expensive nature in energetic terms of the two cellular processes that are needed to perform bone modeling and remodeling. Analysis of mice deprived of insulin signaling in osteoblasts and fed a normal diet also revealed that in true feedback regulation, insulin signaling in osteoblasts hijacks bone cell biology for the purpose of regulating glucose homeostasis. Insulin signaling in osteoblasts favors osteoclast differentiation and, thereby, bone resorption by hampering the expression of *Osteoprotegerin*, a molecule that inhibits the function of the osteoclast differentiation factor RankL (15). As it turns out, this is an efficient way to regulate osteocalcin bioactivity and glucose homeostasis. During the resorption phase of bone (re)modeling, carboxylated osteocalcin present in the bone ECM enters the resorption lacunae where the existing low pH allows for its decarboxylation. Undercarboxylated osteocalcin that is then released can enter the general circulation, act as a hormone, and promote insulin secretion by pancreatic β cells (15).

The study of insulin signaling in osteoblasts also elucidated another, until then, poorly understood aspect of osteocalcin biology, which is why Esp^−/−^ mice that lack a membrane tyrosine phosphatase are a model of gain of function of a secreted molecule like osteocalcin. As mentioned earlier, *Esp* encodes a tyrosine phosphate called OST-PTP that is expressed, as its name indicates, in osteoblasts and Leydig cells of the testes (9). Biochemical investigations showed that a substrate of OST-PTP in osteoblasts is the insulin receptor. As a result of this dephosphorylation, insulin signaling in osteoblasts is then hampered. Conversely, mice that lack *Esp* expression in osteoblasts demonstrate an increase in insulin signaling in that cell type, which results in an increase in bone resorption and an increase in the release of undercarboxylated osteocalcin in the general circulation. Hence, it is because the insulin receptor is a substrate of OST-PTP in osteoblasts that *Esp*-deficient mice are a model of a gain of osteocalcin function (15). A logical consequence of the regulation of bone biology by osteocalcin is that bone is indeed, and as anticipated, a site of insulin resistance in animals fed a high-fat diet (56).

## REGULATION OF STEROIDOGENESIS BY OSTEOCALCIN

**Figure 2:**
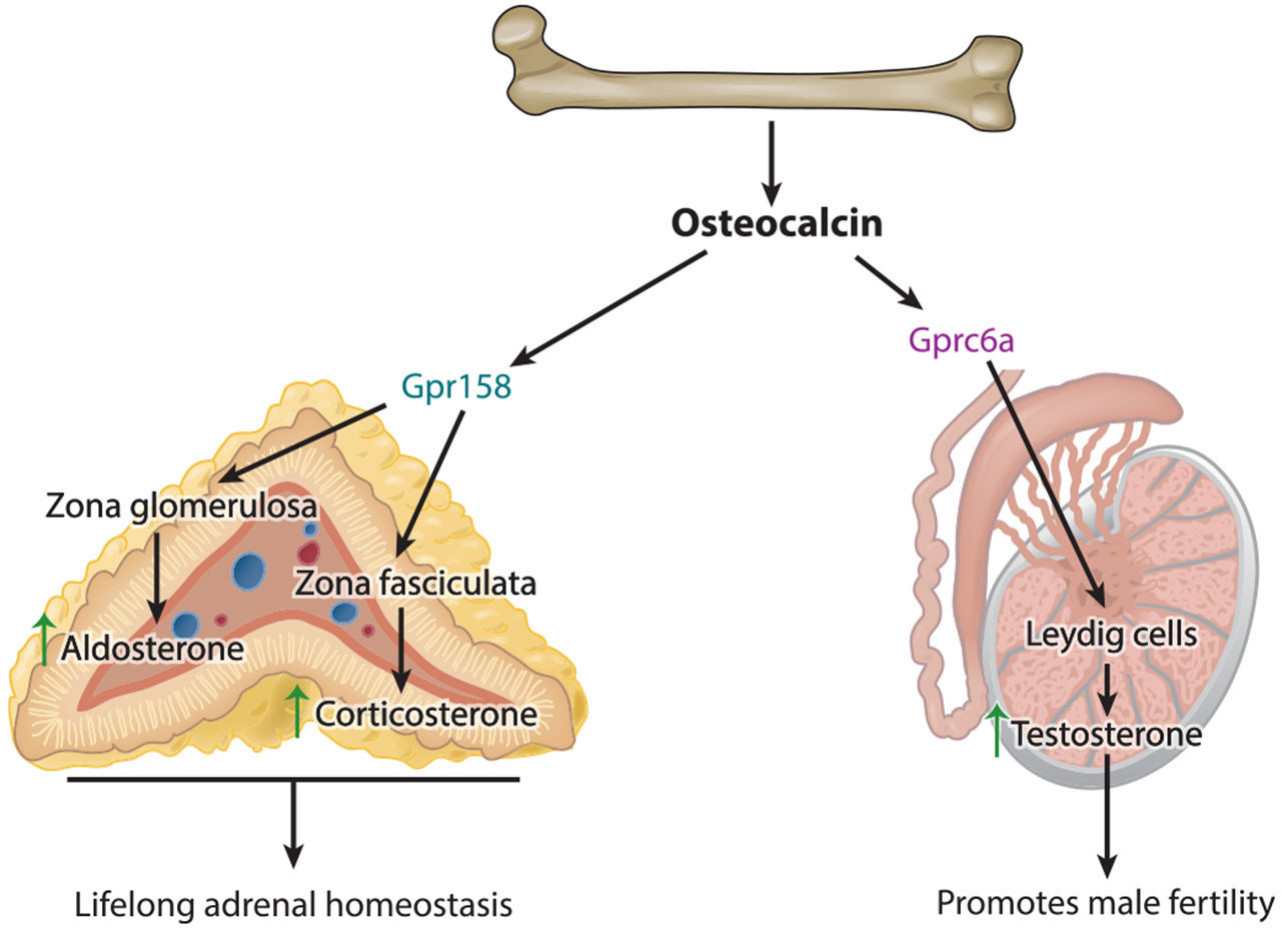
Osteocalcin regulates steroidogenesis in the testes and adrenal glands. In the adrenal gland, cortex osteocalcin acts through Gpr158 and regulates synthesis and secretion of adrenal steroid hormones, aldosterone, and corticosterone. In the testes, osteocalcin acts on the Leydig cells through Gprc6a and increases their proliferation and consequently testosterone production and male fertility.

The initial hypothesis that proposed that bone could be an endocrine organ included the assumption that bone should also regulate reproductive functions. This arm of the original hypothesis was borne out of the founding observation of the entire bone field in clinical medicine, which is that osteoporosis, the most frequent bone degenerative disease, develops mostly in postmenopausal women. This is the reason why it was anticipated that if osteocalcin could regulate reproductive functions, it would be in female animals. Although this was a logical expectation, it was not verified experimentally. Instead, all available evidence, whether obtained in cell culture or in loss- and gain-of-function mouse models, indicates that osteocalcin regulates reproductive functions only in males (Figure 2) (37). In cell culture and in vivo, following its binding to Gprc6a, which is expressed on Leydig cells of the testes but not in any cell types of the ovaries, osteocalcin favors the expression of all the genes necessary for testosterone biosynthesis but does not promote the expression of *Cyp19*, which encodes the aromatase that allows the generation of estrogen from testosterone (37). The fact that mice lacking *Osteocalcin* or *Gprc6a* only in Leydig cells develop hypogonadism in the face of high circulating luteinizing hormone (LH) levels made one important and novel point of physiology: What it meant is that bone via osteocalcin signaling in the testes is a physiological regulator of steroidogenesis in the testes even in the face of normal pituitary functions.

Since the phenotypic abnormalities created by a deficit in osteocalcin signaling in testes are so stereotyped, this function of osteocalcin lent itself to searching whether it also exists in humans. This was of particular interest, since peripheral testicular deficiency is a human condition that recapitulates most, if not all, of the features of reproductive biology seen in mice lacking osteocalcin signaling in the testes: infertility without impotence, low circulating testosterone, high circulating LH levels, and no other known conditions that could affect the biology of the gonads (8). Sequencing osteocalcin and GPRC6A in such patients did not yield any homozygous loss-of-function mutations in osteocalcin but rather identified two patients heterozygous for the same missense mutation in *GPRC6A*. The resulting mutated receptor acts in vitro and in vivo as a dominant-negative mutant (36). Of note, these two patients were also hyperglycemic and glucose intolerant (36). This initial study was followed by several other ones that have identified a polymorphism in GPR6A as a novel risk factor for testicular failure in humans (11, 22).

Together, the ongoing study of the regulation of energy metabolism and of male fertility by osteocalcin signaling verified the validity of the notion that bone is an endocrine organ that influences aspects of every metabolism and reproductive function. It also provided the beginning of an understanding of the signaling mechanisms whereby osteocalcin achieves this function, and it hinted that, as one would expect in mammalian endocrinology, what is true in rodents is true in primates, including humans. However, even if it might have been lost sight of initially, the hypothesis on which this work relied did not exclude the possibility that osteocalcin may have been invented by evolution to regulate other physiological processes besides energy metabolism and male reproductive functions. As the rest of this review article briefly presents, most of the other functions of osteocalcin escape the boundaries imposed by this founding hypothesis but not the notion that bone is a danger repellent. In doing so, and taken at face value, they further increased the regulatory importance of bone in maintaining homeostasis in mammals.

One of the most classic features of osteocalcin biology for the last 35 years has been that *Osteocalcin* expression and *Osteocalcin* circulating levels are decreased by glucocorticoid hormones (GCs), specifically corticosterone in rodents and cortisol in primates (33, 47). In view of the hormonal identity of osteocalcin, these observations raised a novel question: Could it be that, just as is the case for insulin, osteocalcin is a physiological regulator of GC biosynthesis? Testing experimentally and in vivo, this novel hypothesis showed that osteocalcin is necessary for GC synthesis in mice even if they have a normal hypothalamic-pituitary-adrenal axis (Figure 2). Osteocalcin is also sufficient in rodents and in nonhuman primates to increase GC synthesis to the same extent that a similar molar amount of ACTH does (32). This analysis provided more surprises. The first one was that osteocalcin is also necessary and sufficient for proper aldosterone biosynthesis, again in the face of a functional renin-angiotensin system (32). The second one is that, unexpectedly, it is embryo-derived and not postnatal osteocalcin that assumes in an indirect manner the brunt of this function. Embryo-derived osteocalcin is a positive regulator of *Steroidogenic factor1 (Sf1)*, a gene that encodes a transcription factor that is a master regulator of adrenal gland development (32). As a result, embryo-derived osteocalcin signaling in cells of the developing adrenal cortex through another receptor, Gpr158, is a determinant of the formation of the adrenal cortex during embryogenesis and thereby of adrenal steroidogenesis throughout life (32). The physiological outcome of this regulation is that osteocalcin is indirectly a regulator of blood pressure and blood potassium concentration (58).

It is important to note that osteocalcin is so far the only hormone that regulates three distinct steroidogenic pathways that take place in two different organs, the testes and the adrenal glands. If we go beyond osteocalcin, when considering that FGF23 inhibits the synthesis of another steroid hormone, 1α,25(OH)_2_ vitamin D_3_, then bone is a regulator of as many steroidogenesis pathways as the pituitary gland is (23, 45). As mentioned above, the regulation of adrenal steroidogenesis by osteocalcin was not foreseen by the original hypothesis that led to the testing of the endocrine functions of bone. As presented below, the breakdown of the boundaries of the original hypothesis did not stop there. These iterative breakdowns eventually led to a global reappreciation of the functional importance of the skeleton as a regulator of organismal homeostasis.

## OSTEOCALCIN REGULATION OF THE CENTRAL NERVOUS SYSTEM

**Figure 3:**
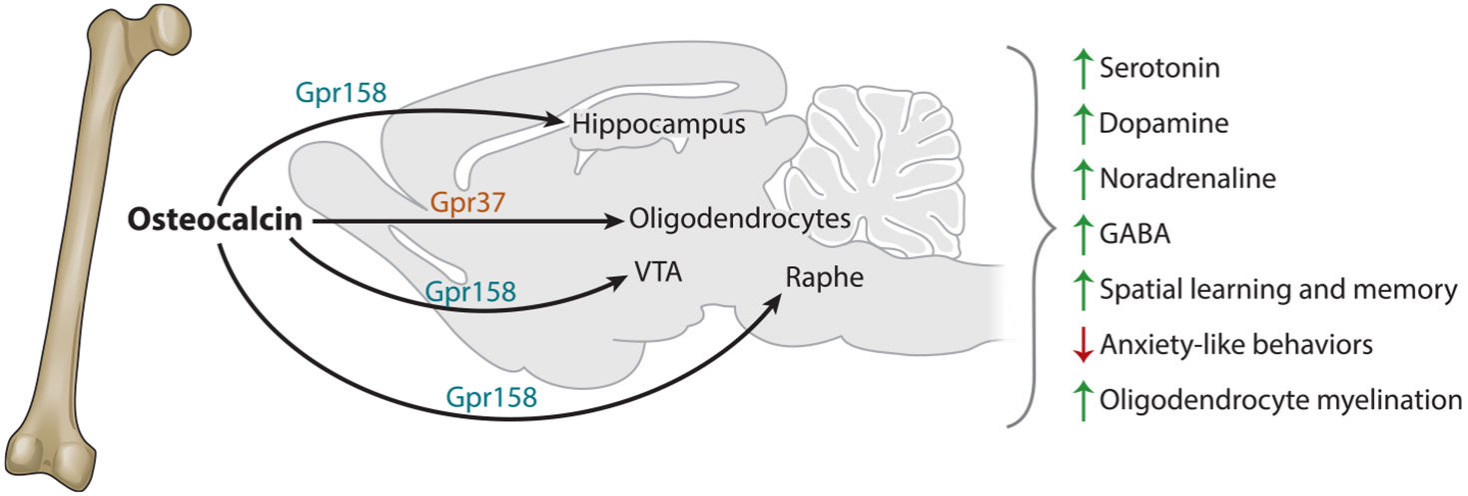
Osteocalcin regulates neural functions. In the brain, osteocalcin acts through two different receptors. Through Gpr37, osteocalcin regulates oligodendrocyte myelination and neural transmission. Through Gpr158, osteocalcin acts on different regions of the brain and regulates synthesis of various neurotransmitters. Through these actions together, osteocalcin regulates spatial learning and memory.

The demonstration at the turn of this century of the existence of a central control of bone mass prompted the following provocative question: Does bone in a feedback manner exert any influence on any brain’s function? (48, 51). A second and more objective reason to test this contention is that *Osteocalcin*-deficient mice demonstrate an abnormal docility that has been noticed by every investigator handling them since we generated and shared them. Since this phenotype was observed in both sexes, this docility could not be explained simply by a deficit in androgen hormones. The availability of *Osteocalcin*-deficient mice allowed us to show that osteocalcin can cross the blood–brain barrier and binds to specific parts of the brain, such as, among others, the CA3 region of the hippocampus, the ventral tegmental area, and the dorsal raphe. Following its binding to another receptor called Gpr158 that is on various neuronal populations in the brain, osteocalcin favors the synthesis of all monoamine neurotransmitters and that of brain-derived neurotrophic factor (BDNF) while it inhibits the synthesis of the inhibitory neurotransmitter GABA (3). In addition, osteocalcin signals in oligodendrocytes through yet another receptor, Gpr57, and regulates in these cells their ability to myelinate the central nervous system (37) (Figure 3). The functional consequences of these cellular and molecular events are that osteocalcin prevents anxiety and depression and favors spatial learning. Here again, osteocalcin not only is necessary to prevent anxiety and increase cognition but also is sufficient to restore the memory deficit and dampen the anxiety and depression that is otherwise observed in older mice that, again, have low circulating osteocalcin levels. Evidence from multiple experiments showed that this is also, in part, only a developmental function since maternal osteocalcin crosses the placenta and acts in the brain of embryos to promote brain development (3).

## OSTEOCALCIN REGULATION OF THE AUTONOMIC NERVOUS SYSTEM ACTIVITY

**Figure 4:**
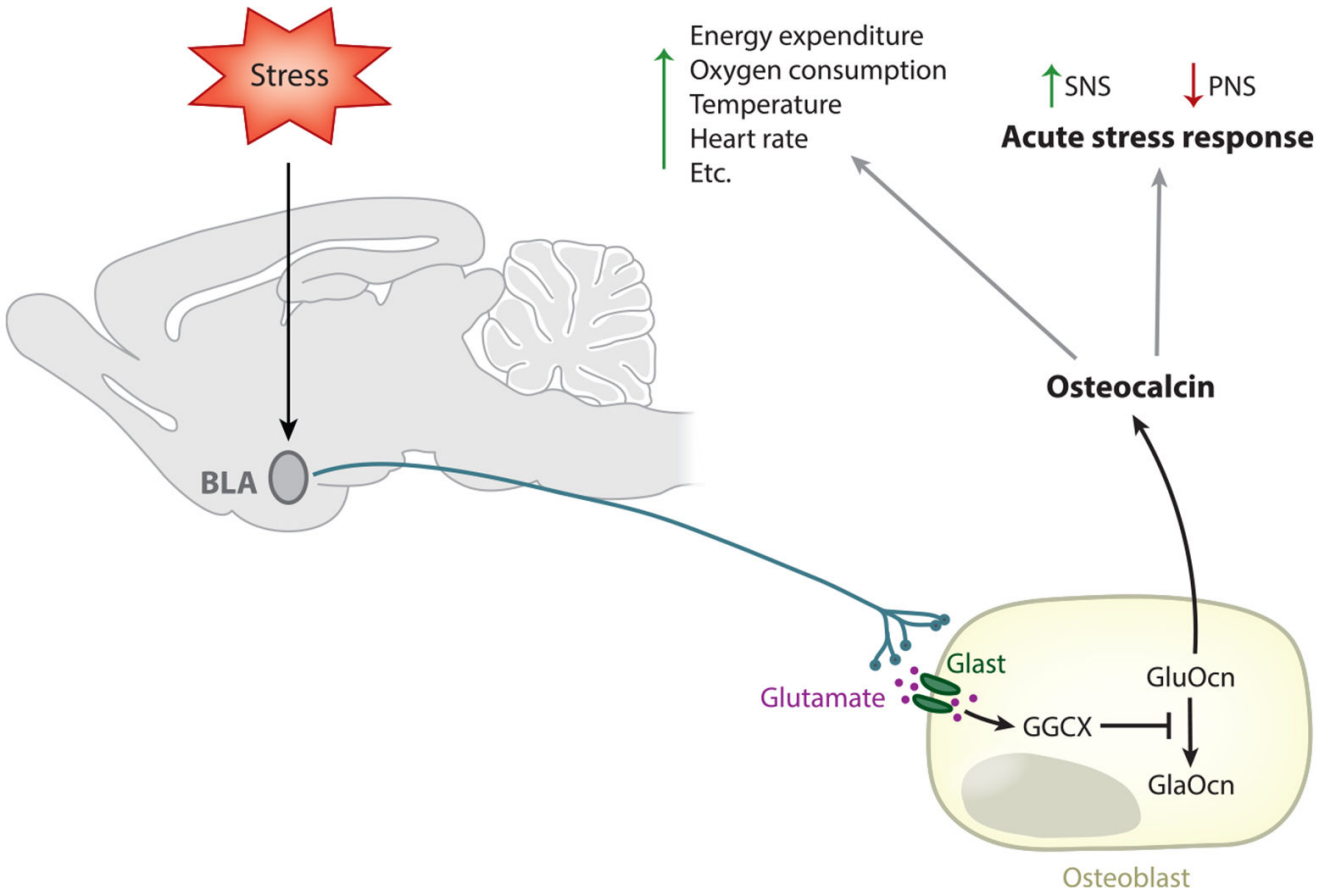
Osteocalcin regulates the acute stress response. Osteocalcin is released during acute stress and affects parasympathetic neuronal activity. Glutamate released at the neural-osteoblast junctions is taken up by osteoblasts wherein it inhibits the enzyme GGCX that is responsible for the inactivation of osteocalcin by converting Glu to Gla residues. This leads to an increase in the release of active osteocalcin that increases sympathetic, and concomitantly decreases parasympathetic, nervous system activity. Abbreviations: BLA, basolateral amygdala; PNS, parasympathetic nervous system; SNS, sympathetic nervous system.

The last out-of-the-hypothesis function of osteocalcin we need to mention was uncovered in an effort to find a common purpose for all functions of osteocalcin. In trying to do so, it appeared plausible, if not likely, that just as its host organ does through other means, osteocalcin may favor escaping from danger (Figure 4). It constantly does so through its ability to promote the memory of where the predator or the food could have been 1 h or 1 day ago. Osteocalcin also favors escaping danger through its ability to favor exercise, which is needed to escape danger. If this interpretation of a common thread between the different functions of osteocalcin is correct, then osteocalcin could or should be needed for the development of the quintessential physiological process needed to escape danger, the acute stress response.

In vivo testing of the latter hypothesis showed that circulating osteocalcin levels rise within minutes in mice, rats, and humans exposed to a stressor, as stress hormone levels should (3). This effect was specific, as no other osteoblast-enriched proteins saw their circulating levels increase following exposure to stressors (3). Osteocalcin fulfills the second criterion of a stress hormone, as signaling is needed for a stressor to induce an acute stress response (3). In brief, stressors signal in the brain in the basolateral amygdala and other centers of fear in the brain (27). These neurons use glutamate as a neurotransmitter that enters the osteoblast through the transporter Glast and exerts a competitive inhibition of the enzyme gamma carboxylase, the one that is responsible for the carboxylation and inactivation of osteocalcin (3). As a result, uncarboxylated or undercarboxylated osteocalcin can be released into the general circulation during the acute stress response (3). Osteocalcin then binds to Gprc6a, which is expressed on postganglionic parasympathetic neurons, inhibits acetylcholine synthesis, and reuptakes. Thus, the sympathetic tone can increase unabated and the acute stress reaction can unfold (3).

## COHERENCE AMONG THE FUNCTIONS OF OSTEOCALCIN

Coming to the end of this long description of the physiological functions that are regulated by osteocalcin signaling, there are objective facts that emerge; these facts bring us back to a notion alluded to in the preamble of this review article. As one would expect, these facts also bring up novel questions. Chief among the emerging facts is that bone is indeed an endocrine organ regulating multiple aspects of organismal physiology ranging from cognition to reproduction. A second fact that was unexpected is that osteocalcin often acts indirectly and rather as a regulator of regulatory molecules. Indeed, it is upstream of the synthesis of insulin, testosterone, adrenal steroid hormones, many neurotransmitters in the brain and in the periphery, etc. As a result of these multiple regulations of regulatory molecules, osteocalcin regulates glucose homeostasis in multiple ways, affecting physiological functions including male fertility, the acute stress response, exercise, adrenal steroidogenesis, and blood pressure, to name only a few. This very enumeration suggests that, possibly because of the high energetic cost of its physiology and possibly also for other not-yet-identified reasons, bone has been endowed by evolution with the ability to act as a rheostat of many physiological processes. Taken at face value, this alone is a rich development of bone biology.

Going through the long list of regulatory functions regulated by osteocalcin, this investigation brought to bear that osteocalcin—and, more broadly, bone—regulates a larger number of physiological functions than anticipated. This wealth of regulatory functions fulfilled by a single hormone begs the question of what could be a common thread between these functions. Looming behind this question is another one, broader and less amenable to experimental manipulation: What may have been the original purpose of bone when it was invented by evolution as animals left the sea to move to land? Although there is apparently no clear link between cognition, muscle function during exercise, and glucose mobilization, as presented above, a broader view of these functions and especially of their outcomes suggests that there may be a commonality between them. Again, as mentioned above, cognition is needed to remember where the predator was and where the food was, and without this function, life in the wild would be very dangerous. Even if an animal has an excellent memory, if it cannot increase its muscle function to run, and/or if it cannot mobilize glucose, its ability to survive in the wild is limited. In this light, many physiological functions that are regulated by osteocalcin, including, of course, the initiation of the acute stress response, are needed to sense and escape danger. As a matter of fact, and to get back to the preamble of this review article, the purpose of sensing and overcoming danger is shared between the endocrine and the structural and ambulatory function of bone. The lengths to which bone goes to fulfill this function, through both endocrine and structural means, provide the image of a tissue that has conferred a considerable evolutionary advantage to bony vertebrates.
